# Locomotor Anatomy and Behavior of Patas Monkeys (*Erythrocebus patas*) with Comparison to Vervet Monkeys (*Cercopithecus aethiops*)

**DOI:** 10.1155/2013/409534

**Published:** 2013-09-26

**Authors:** Adrienne L. Zihlman, Carol E. Underwood

**Affiliations:** University of California, Santa Cruz, Social Sciences 1, 1156 High Street, Santa Cruz, CA 95064, USA

## Abstract

Patas monkeys (*Erythrocebus patas*) living in African savanna woodlands and grassland habitats have a locomotor system that allows them to run fast, presumably to avoid predators. Long fore- and hindlimbs, long foot bones, short toes, and a digitigrade foot posture were proposed as anatomical correlates with speed. In addition to skeletal proportions, soft tissue and whole body proportions are important components of the locomotor system. To further distinguish patas anatomy from other Old World monkeys, a comparative study based on dissection of skin, muscle, and bone from complete individuals of patas and vervet monkeys (*Cercopithecus aethiops*) was undertaken. Analysis reveals that small adjustments in patas skeletal proportions, relative mass of limbs and tail, and specific muscle groups promote efficient sagittal limb motion. The ability to run fast is based on a locomotor system adapted for long distance walking. The patas' larger home range and longer daily range than those of vervets give them access to highly dispersed, nutritious foods, water, and sleeping trees. Furthermore, patas monkeys have physiological adaptations that enable them to tolerate and dissipate heat. These features all contribute to the distinct adaptation that is the patas monkeys' basis for survival in grassland and savanna woodland areas.

## 1. Introduction

Patas monkeys (*Erythrocebus [Cercopithecus] patas*) live in dry, seasonal habitats in grass and woodland savannas across northern Africa between the equator and Sahara, from Ethiopia to Senegal and southwards into northern Tanzania [1]. Their adaptation to these landscapes contrasts with that of sympatric vervet monkeys (*Cercopithecus aethiops*) and baboons (*Papio cynocephalus*) [2]. The discovery that patas monkeys run at high speeds was interpreted as a unique locomotor adaptation to avoid predators [3, 4]. Skeletal features—lengthened limbs, short digits, and digital foot postures—have been cited as the anatomical bases (e.g., [3, 5–7]). Compared to New World monkeys (Ceboidea) and apes (Hominoidea) Old World monkeys (Cercopithecoidea) are remarkably uniform in body dimensions and share a “generalized quadrupedal” locomotor pattern [8]. Due to anatomical similarities, we incorporate soft tissue associated with the locomotor system to tease out potential species-specific patterns.

Two studies stand out in adding new details to patas locomotor function. Based on dissections of the leg and foot and radiography and cine-film, Wood [9] linked the musculoskeleton of the hindlimb with locomotor biomechanics. Compared to baboons, patas monkeys have reduced motion at the ankle joint, longer tarsals and metatarsals, shorter phalanges, and fore- and hindlimbs oriented in the parasagittal plane. Wood interpreted this anatomy as convergent with cursorial mammals. In a later study, Hurov [10] compared patas and vervet monkey vertebral columns. Using cine-film, he measured the flexibility of the back during fast running and found that the patas vertebral column has limited flexion-extension compared to that of vervets. He found that vervets have thicker intervertebral discs and a broader transverse rib cage that promotes greater back mobility and a significantly increased stride length. Patas monkeys have thinner intervertebral discs and a narrower and deeper rib cage that decreases sagittal bending; patas monkeys rely on lengthened fore- and hindlimbs for increased stride length. These regional studies clarify the role of leg, foot, and back structures in locomotor function. To date there have been no studies of complete patas locomotor anatomy, including forelimbs as well as hindlimbs, and musculature as well as the skeleton.

The research presented here adds to previous studies and provides new data on functional anatomy of patas with comparisons to vervet monkeys. Dissections of entire animals give holistic information on limbs, back, tail, and musculoskeletal system. Vervet monkeys serve as a useful comparison because (1) they represent a generalized quadruped; 15 million year old fossil monkeys, *Victoriapithecus*, most closely resemble vervet body size and limb bone traits [11, 12]; (2) they are the closest relatives to patas with a recent divergence of 2–4 million years ago [13–15]; (3) they are sympatric with patas at study sites so that behavioral ecologies can be compared.

We address the following questions: how unique are patas monkeys and how have they diverged from vervet monkeys in limb proportions, muscle distribution, and muscle groups? How well do these data support the concept that patas monkeys converge with cursorial mammals? Given the close evolutionary relationship of patas and vervet monkeys, we expect patas to show overall anatomical similarity to vervet monkeys. We also expect that differences that do exist will be slight, but when taken together will show a distinct functional pattern.

## 2. Materials and Methods

### 2.1. Animal Sample

Two patas monkeys from the University of California, Berkeley colony for research on social behavior died of natural causes and became available for postmortum study. The vervet monkeys were acquired for teaching and donated to this study.All animals were dissected unpreserved (Table 1).

The male patas was a well-muscled adult with fully erupted canine teeth and all long bones fused; he showed no sign of illness or disease. His body weight at 11.3 kg is in the range reported for captive and wild males (e.g., 7.4–12.6 kg, [3, 10, 16, 17]). The female patas was immature (1.5 yr old) and at 4.1 kg is close to but not yet at adult weight (e.g., 4.4–7.6 kg, [10, 17]), an indicator of how quickly female patas monkeys mature [18].

The vervet monkeys were well-muscled adults. The male weight at 6.4 kg is in the range of published reports (4.3–6.6 kg, [10, 17, 19]) and the female at 3.5 kg similarly (2.9–5.8 kg, [17, 19]). Only the males are compared in muscle distribution and proportions to avoid the confounding variable of sex, and in the case of the patas female, age differences [19, 20].

### 2.2. Methods of Dissection and Data Collection

Prior to dissection, photographs and external measurements (after [21]) are taken on head/trunk length (vertex to ischium) and tail length. Standardized dissection methods provide quantitative information on body segments, body composition, and muscle groups [22–24].

**Figure 1:**
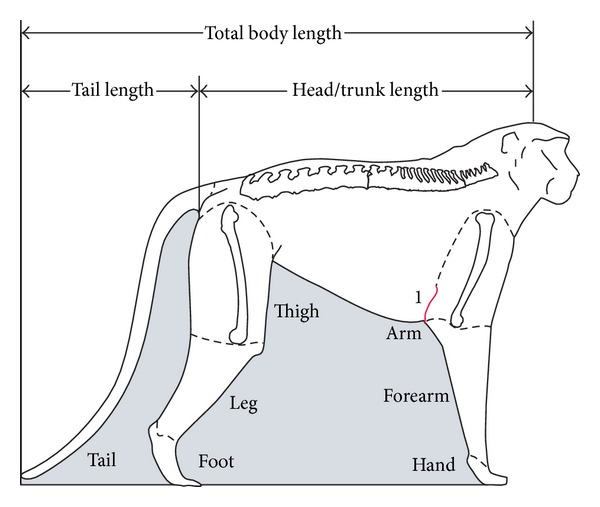
External measurements; body and limb segments; 1 indicates the axial skin fold relative to distal humerus on the patas monkey.

One side of the body is dissected by segments. The forelimb is detached from the trunk at the shoulder joint, the hindlimb, and at the hip joint. The tail is weighed as a unit. Each limb is then separated into arm, forearm, and hand and thigh, leg, and foot segments. Each is weighed and further divided into muscle, bone, and skin; each tissue is weighed separately and recorded with its associated segment (Figure 1).

On the other side, individual muscles are dissected with tendon intact and weighed immediately. Skin, bone, and other tissues are separated and weighed.

Similarly, the head/trunk muscles, including the back extensors and tail muscles, are separated and weighed.

After the skeleton is cleaned using dermestid beetles, the bones are measured.

### 2.3. Methods of Analysis

(1) Segment mass analysis on the forelimb, hindlimb, and tail masses are each calculated relative to total body mass; segments within each limb are calculated relative to total limb mass. 

(2) Body composition is determined by adding all the muscle, bone, and skin tissues from the entire dissection. Each is then calculated as a percentage of total body mass. 

(3) Total body bone is taken as 100%. Its distribution to the body is determined as follows. Forelimb: humerus, radius, ulna, and hand bones from both sides of the body are taken as a percent of total body bone; similarly in the hindlimb, femur, patella, tibia, fibula, and foot bones are taken as percent of total bone. Bone from the tail is calculated separately. Skull, trunk, pectoral, and pelvic girdles comprise the head/trunk segment.

Total body muscle is taken as 100%. Its distribution to limb and tail segments is determined as follows. Forelimb: all forelimb muscles plus those that attach to the humerus (latissimus dorsi, pectoralis major, teres major, teres minor, supraspinatus, infraspinatus, and subscapularis) are added together, doubled, and taken as a percent of total body muscle. Similarly, all hindlimb muscles plus hip muscles that attach to the femur (the gluteals, iliopsoas, obturator internus and externus, and gemelli) are taken as a percent of total body muscle. Tail muscle is taken as a percent of total body muscle.

(4) Muscles are grouped according to their relationship to movement, and a ratio of antagonists is determined, for example, flexors to extensors. Relative mass of individual muscles or muscle groups serves as a gross approximation of functional importance at the joint. Only the adult male patas and male vervet are compared in limb segment, muscle distribution, and muscle mass due to possible confounding variables of age and sex in these dimensions (e.g., [20, 25, 26]).

(5) Indices are calculated from bone lengths: claviculohumeral index (clavicle as percent of humerus length, after [27]); humerofemoral (humerus to femur length); intermembral (humerus plus radius as a percent of femur plus tibia lengths); brachial (humerus to radius length); crural (femur to tibia length). Foot bones are measured, and relative lengths are calculated after Schultz [28]. Relative tail length is taken as a percentage of total body length (trunk length plus tail length).

## 3. Results

External assessment of the body is made prior to dissection. The patas chest is narrow and deep which is reflected in the lowest chest index (84) reported for Old World monkeys by Schultz [29]. The skin fold at the axillary region attaches low on the arm segment to bring the forelimb close to the trunk (Figure 1).

The male patas tail is 47.4% of total body length, the male vervet is 59.2% and female vervet 58.8%. These measurements were not available for the female patas.

The contribution of muscle, bone, and skin, components of the locomotor system is reported in Table 2. Both the female patas and the female vervet have relatively less muscle mass than do the males, a sex difference noted in some Old World monkey species [19, 20]. These three tissues together comprise 68.1 to 74.7% of total body mass.

**Figure 2:**
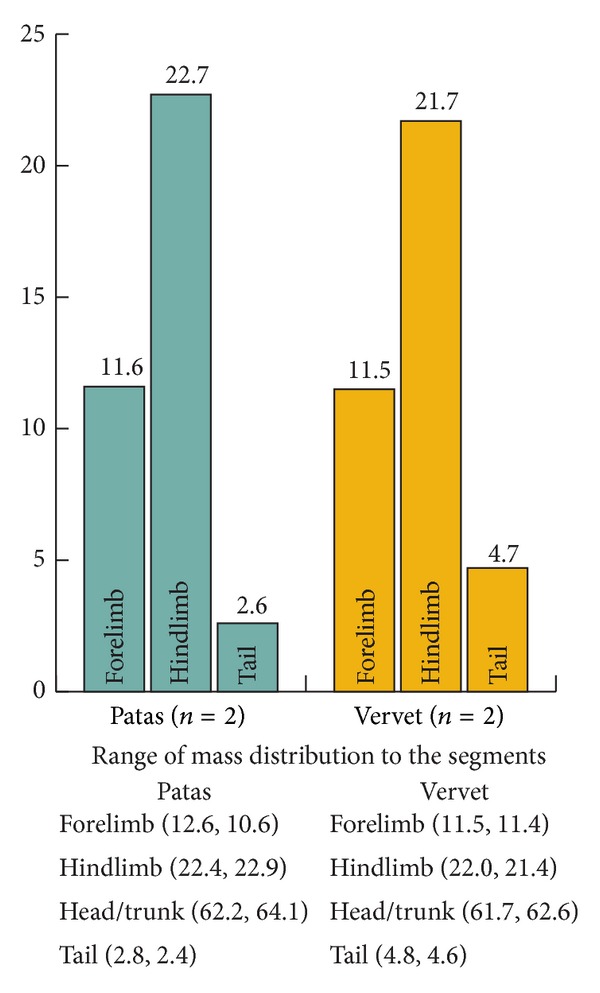
Average mass distribution as % total body mass.

**Figure 3:**
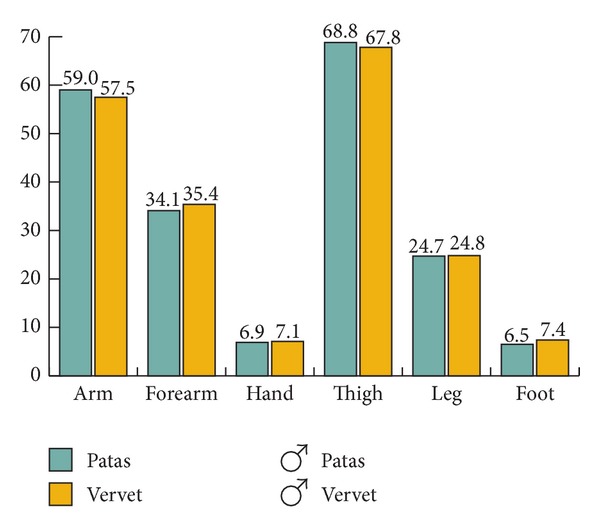
Mass distribution as % total limb mass; males only.

In relative mass of body segments, the two species are similar, although the patas monkeys have a slightly heavier head/trunk segment and lighter tail. The relatively lighter forelimb of the female patas may be due to her younger age or to being female (Figure 2). Within the segments, patas monkey limbs are slightly more tapered, with heavier arm and thigh segments and lighter hand and foot (Figure 3).

**Figure 4:**
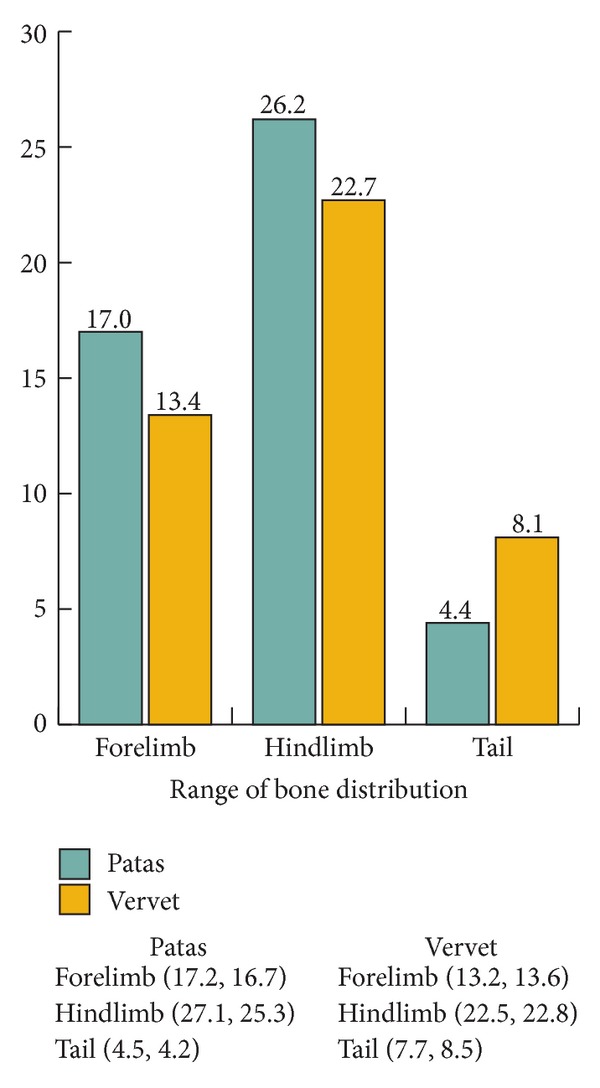
Average bone distribution as a % of forelimb, hindlimb, and tail.

Distribution of bone to the segments is the same in the female and male of each species, and the two species differ in that patas monkeys have relatively heavier bones (Table 2, Figure 4).

**Figure 5:**
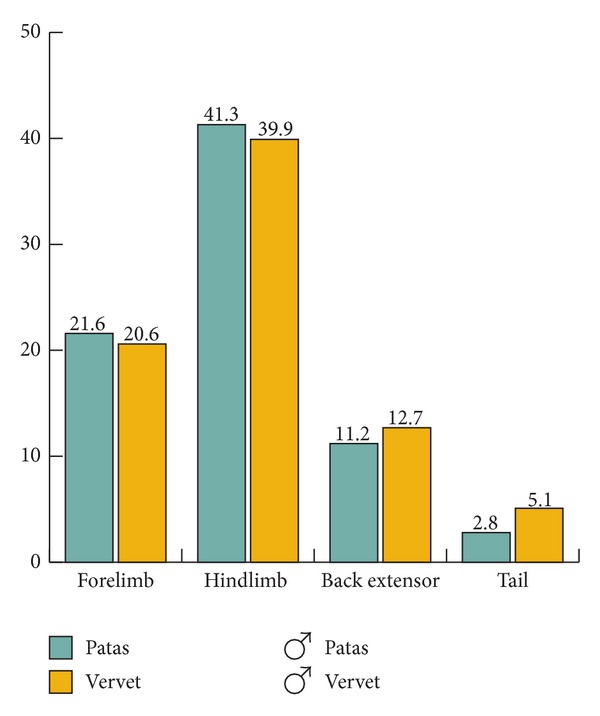
Distribution of muscle to segments as a % of total body muscle; males only.

Distribution of muscle to the segments is similar in the male patas and male vervet, though the patas has slightly more muscle in the hindlimb and less in the back and tail (Figure 5).

**Figure 6:**
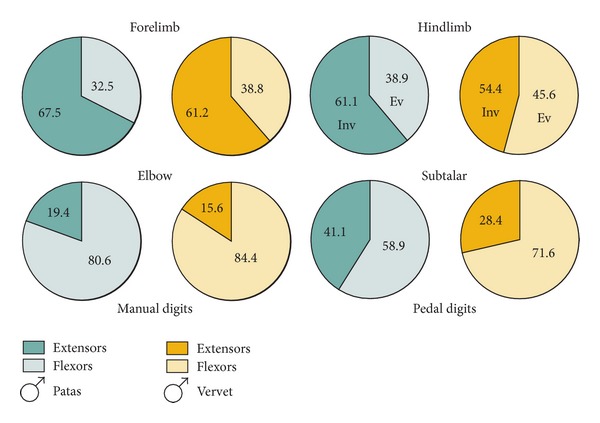
Ratios of the most distinctive muscle groups within the limbs, males only.

Muscle groups at key joints show differences. Patas and vervet differ in relative proportions of extensors to flexors at the elbow joint and extrinsic digital extensors and in the hindlimb, plantar flexors (ankle extensors), subtalar invertors, and extrinsic digital extensors (Table 3, Figure 6).

The indices calculated from bone lengths are shown in Table 4. The high humero-femoral index indicates that the forelimb and hindlimb are similar in length, whereas the lower index in vervets reflects a shorter humerus and longer femur. The intermembral shows a pattern similar to that of the humero-femoral index. The brachial and crural indices indicate that the radius and tibia are somewhat longer relative to the humerus and femur, respectively, than in the vervets. The claviculohumeral index reflects the narrow chest breadth and the closer approximation of the shoulder joints in patas.

**Figure 7:**
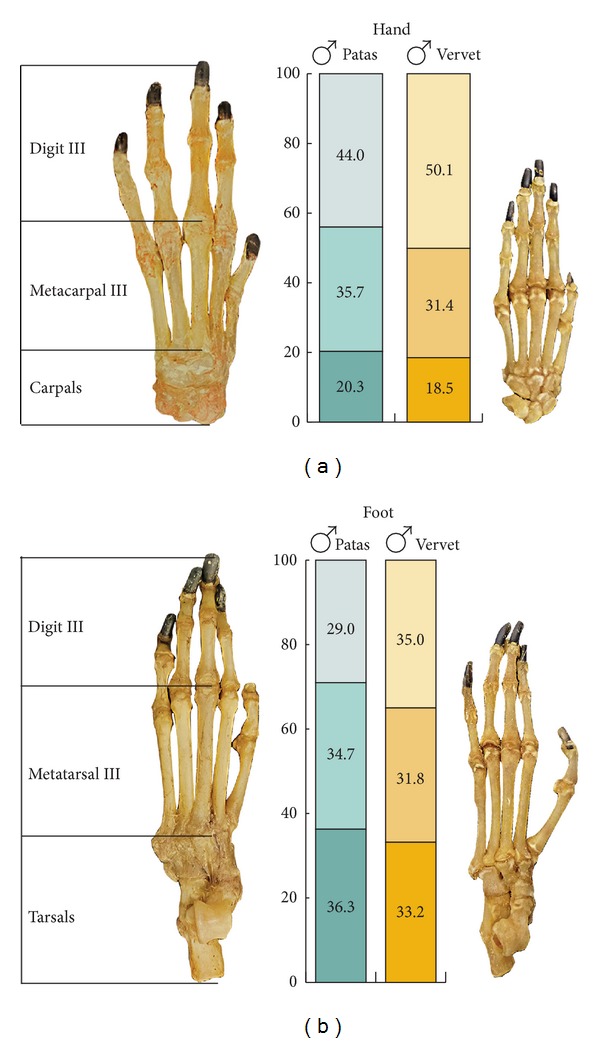
Hand segments as % total hand length (a); foot segments as % total length (b). Hands and feet scaled.

Patas hands and feet have longer carpal and tarsal regions, longer metacarpals and metatarsals, and shorter digits compared to vervets (Figure 7).

## 4. Discussion

What does this comparative approach with the addition of soft tissue and body proportions reveal about the uniqueness of patas monkey locomotor anatomy and behavior? Remarkably, even with notable differences in body mass, patas monkeys are built on the same plan as other Old World monkeys. Body proportions, for example, forelimb mass (11–13%) and hindlimb mass (20–24%), are not only characteristic of vervets [30] but also of macaques (*Macaca fuscata* and *M. mulatta*, [22, 25]), baboons (*Papio cynocephalus*, [9]), and langurs (*Semnopithecus entellus*, [31]), with variation among species in tail proportions. The similar body plans reflect a shared anatomy that underpins Old World monkey quadrupedal locomotion. Patas and vervet monkeys also share similar body compositions and distributions of bone and muscle mass. Hence, the addition of soft tissue data supports and adds to Schultz's general conclusions on structural uniformity of Old World monkeys. 

However, small differences in the patas body shape affect locomotor function. Patas limbs are aligned under the trunk to ensure movement in the parasagittal plane. This alignment is achieved by a combination of vertically oriented scapulae against a narrow and deep chest, shoulder joints close together indicated by short clavicles, and attachment of the skin that binds the arm segment close to the trunk. These features reduce abduction of the limbs and keep them functioning in the fore and aft plane.

Limb length proportions also affect body shape. Patas locomotion depends upon a long stride and relatively efficient gait. The relatively longer radius and carpals and metacarpals and a digitigrade hand posture lengthen the forelimb. The slightly lengthened tibia and tarsals and metatarsals, and a digitigrade foot posture, all contribute to a longer hindlimb. The combination of forelimb and hindlimb lengths function to increase stride. The lighter hand and foot segments reduce the force needed during recovery phase of the stride.

The distribution of limb mass and muscle proportions affects power and mobility. The proximal shift of muscle to the shoulder and hip joints is reflected in a somewhat greater mass of arm and thigh segments, compared to vervets. The shorter and lighter tail in patas also keeps body and muscle mass closer to the hip joints. Proportions of muscle mass suggest greater propulsive action. Heavier elbow extensors, triceps brachii, and manual digital extensors underscore extension and deemphasize flexion during recovery phase of gait. Lighter back extensor muscles correlate with the less flexible back in patas [10].

Plantar flexors, or ankle extensors, (gastrocnemius, soleus, and plantaris) are five times the mass of the dorsiflexors (tibialis anterior) in patas compared to vervets' ratio of three. In addition to power at the knee joint, the plantar-flexors assist in maintaining a digitigrade foot posture and along with heavier digital extensors of the toes reflect the absence of a plantigrade foot posture during walking [9]. By cine-film and radiography Wood showed that the patas foot moves little when the lower limb is suspended during recovery, unlike the baboon's foot which inverts during recovery. Patas foot invertor muscles are relatively heavy and may compensate for the bony configuration at the ankle by maintaining joint mobility for climbing. The slender digits are straight and short, and the sole narrow with the thickest cushion of fat over the metatarsal heads [9].

How well does patas monkey anatomy fit with the notion of convergence with cursorial mammals? Cursors are generally defined by speed [32], and it was the ability of patas monkeys to run fast that first called attention to this aspect of their locomotion. Features of cursorial mammals include a deep thorax to accommodate a long vertically oriented scapula, narrow shoulder breadth often with reduced or absent clavicles, long limbs relative to trunk length, long and light distal segments, digitigrade posture, and muscle mass concentrated at the shoulder and hip joints [22, 32]; however, animals that have these features are not necessarily cursorial [33].

Patas monkey anatomical features have shifted somewhat in this direction. The digitigrade foot posture is perhaps the most noted. However, the hand is also digitigrade in walking but becomes palmigrade in running [34, 35]. The convergence of patas with cursorial mammals is slight because of the need to retain limb joint mobility, particularly in the forelimb, which is needed for climbing, manipulation during foraging and catching insect prey, and grooming. Overall, the comparison is superficial, and what predominates is the primate heritage of patas as Old World monkeys and particularly as guenons (e.g., [36, 37]).

The locomotor behavior of patas monkeys has been of interest since Hall's field study in Uganda called attention to their ability to run fast, presumably to avoid predators. Later research at sites in Cameroon (e.g., [38–40]) and Kenya (e.g., [41–43]) provided a detailed picture of behavioral ecology of patas monkeys and comparisons with sympatric vervet monkeys. Patas monkeys have a variety of means to cope with predators in addition to running. In the grasslands, with predators such as jackals, hyenas, lions, cheetahs, and wild dogs, patas monkeys depend upon vigilance from tall trees, giving and responding to alarm calls, crypticity, and rapid flight into the safety of woodlands; at night when leopards hunt, patas monkeys disperse into several sleeping trees and avoid using the same trees in consecutive nights when arboreal predators are present [3, 18, 39–41, 44].

Patas monkeys utilize grasslands and open acacia woodlands but avoid riverine woodlands, whereas vervet monkeys and baboons prefer woodlands and riverine edges [3, 39–41]. The majority of patas daily activities are spent on the ground in search of food. Field researchers emphasize the long distances patas monkeys travel on the ground and characterize their locomotion as one of continual walking rather than running [40, 45–47]. Patas monkeys cannot digest fiber-rich foods of herbaceous plants as most species do that inhabit the savanna. Their grassland foods are small, highly nutritious items like grasshoppers, ants, acacia gums, and seeds which are dispersed with low density and therefore require more time to cover a wide area each day [42, 47–50]. Patas groups are mobile throughout the day and forage for insects as they walk. They stand bipedally to gnaw and scrape gum and collect ants from tree trunks and branches [41]. Their diet yields substantial amounts of energy, protein, and minerals from acacia gum and *Crematogaster* ants [43, 48]. In the dry season patas monkeys can expand their range to include areas with water and therefore are not limited to where water is readily available [40]. Furthermore, patas monkeys have enhanced heat tolerance due to physiological properties of the skin. A higher density of eccrine sweat glands on the chest and larger though less numerous glands on the lateral thigh than rhesus monkeys result in higher sweat rates, which are more efficient at dissipating heat [51–53]. 

In summary, compared to vervet monkeys and baboons, which overlap in some areas of the patas habitat, patas monkeys have larger home ranges and longer daily travel and spend more time moving and foraging. Vervets spend less time moving, more time resting, eat less animal matter, focus more on fruits, flowers, leaves, and lipid-rich seeds. They spend more time feeding in trees than on the ground and in the dry season are confined to readily available water sources in their immediate home range. The way that patas monkeys live, move, and exploit resources that occur in savanna habitats is unique among African monkeys and contrasts with the behavior of the smaller vervets and the larger baboons.

## 5. Conclusions

The patas adaptation involves anatomical structures of muscle, bone, and skin devoted to the locomotor system, which together make up about 70% of body mass. Their skin is multifunctional. Not only does the skin cover the musculoskeleton, but it mechanically restricts the forelimbs to the parasagittal plane. Physiologically it contains sweat glands that dissipate heat and contribute to heat tolerance in environments with less continual tree cover where patas monkeys travel.

Efficient long distance walking is enhanced through a mosaic of musculoskeletal characters, including body shape, limb length, joint orientation and movement, and muscle function. Long legs and digitigrade foot posture are key features but so are changes in the forelimb. Their locomotor abilities underpin and support their adaptation to an unusual Old World monkey ecology and allow patas to forage effectively for insects, seeds, and gums as they move widely across their range between these small packets of dispersed food and water sources.

The emphasis on patas locomotion—or baboon or vervet locomotion—is not just one of “terrestriality” but of utilizing life on the ground in a particular, species-specific way. Taking into account the entire animal, its structures, and their functions as they are integrated into the totality of their behavior in the environmental context provides a more complete analysis of the unique adaptation of this unusual monkey species.

## Figures and Tables

**Table 1:** Sample.

Animal	Age/sex	Body mass (g)	Trunk length (cm)	Tail length (cm)
*Erythrocebus patas *	Adult male	11260.0	65.5	59.0
*Erythrocebus patas *	Immature female	4088.5	na	na
*Cercopithecus aethiops *	Adult male	6450.0	49.0	71.0
*Cercopithecus aethiops *	Adult female	3500.0	42.0	60.0

**Table 2:** Body composition. Percent contribution of major tissues to total body mass.

Animal	Body mass (g)	Muscle	Bone	Skin
Patas male	11260.0	44.2	15.2	11.3
Patas female	4088.5	39.7	14.7	13.7
Vervet male	6450.0	48.0	11.1	12.3
Vervet female	3500.0	42.9	12.4	12.9

**Table 3:** Individual muscle weights to the nearest tenth of a gram.

Forelimb muscles	Patas		Vervets	
	Male	Female	Male	Female
Trunk to humerus				
Pectoralis major	44.5	17.0	32.6	13.1
Latissimus dorsi	55.6	19.0	46.0	18.9
Teres major	17.9	8.0	12.7	5.0
Teres minor	4.5	1.7	2.3	1.3
Subscapularis	29.8	11.0	21.5	10.6
Infraspinatus	23.5	8.5	13.1	7.5
Supraspinatus	20.9	7.4	11.6	6.1
Arm				
Deltoid	34.3	12.2	23.6	10.9
Biceps brachii: long	35.1	15.1	28.5	9.9
Biceps brachii: short	w/long hd	w/long hd	w/long hd	2.2
Coracobrachialis	1.6	1.5	1.0	0.5
Brachialis	18.0	8.2	6.9	4.0
Triceps: long	49.2	20.6	32.7	15.8
Triceps: lateral	39.8	18.6	24.2	12.3
Triceps: medial	28.0	11.5	12.8	7.7
Dorsoepitrochlearis	5.8	3.4	6.4	3.0
Forearm				
Brachioradialis	6.0	2.9	12.9	4.5
Palmaris longus	4.4		2.0	1.4
Flexor carpi radialis	6.4	1.9	3.2	1.6
Flexor carpi ulnaris	14.0	5.5	8.7	6.1
Flexor digitorum superficialis	8.8	3.1	7.1	3.7
Flexor digitorum profundus	29.2	11.7	18.0	9.8
Flexor pollicis longus	w/profundis	0.1	w/profundus	w/profundus
Supinator	2.6	1.6	1.4	1.1
Pronator teres	7.6	3.3	5.3	2.4
Pronator quadratus	1.6	0.5	0.9	0.5
Extensor carpi radialis longus	6.8	2.8	5.6	2.8
Extensor carpi radialis brevis	7.8	3.2	4.9	2.3
Extensor carpi ulnaris	4.8	2.1	2.4	1.5
Extensor digiti minimi	1.6	0.9	1.3	0.5
Extensor digiti communis	6.8	3.0	2.7	1.7
Extensor indicis	0.7		0.6	0.4
Anconeus		0.3	0.4	
Abductor pollicis longus	4.0	1.6	1.5	0.5
Extensor pollicis longus	0.9	0.5	0.5	0.3
Extensor pollicis brevis			0.9	1.0

Hind limb muscles	Patas		Vervets	
	Male	Female	Male	Female

Hip				
Gluteus maximus	44.8	15.0	23.4	9.5
Tensor fascia latae	w/glut max	7.0	6.8	3.1
Gluteus medius	114.3	42.6	50.0	27.9
Piriformis	w/glut med	1.9	w/glut med	w/glut med
Gluteus minimus	9.4	4.1	5.0	3.2
Gemelli	3.4	1.6	1.8	1.0
Obturator externus	16.5	3.6	6.9	4.6
Obturator internus	12.6	5.6	7.3	**5.0**
Quadratus femoris	2.9	3.5	5.1	2.8
Iliopsoas	68.9	25.4	57.1	20.1
Psoas minor	11.0		w/iliopsoas	w/iliopsoas
Tail muscles total	136.5	59.0	157.8	90.7
Thigh				
Sartorius	5.0	1.6	4.9	2.4
Gracilis	37.2	16.5	20.9	9.8
Pectineus	7.0	w/mag	5.9	2.8
Adductor magnus	108.9	37.9	66.3	40.9
Adductor longus	7.0	w/mag	5.6	w/mag
Adductor brevis and minimus	20.8	w/mag	7.7	w/mag
Rectus femoris	31.5	12.7	28.7	14.5
Vastus lateralis	92.7	60.0	54.9	31.6
Vastus intermedius	26.6	w/lat	10.8	4.6
Vastus medialis	23.6	w/lat	19.0	10.3
Biceps femoris: long	119.8	56.6	71.8	31.7
Biceps femoris: short	w/long hd	w/long hd	w/long hd	w/long hd
Semimembranosus	32.3	42.8	29.3	12.3
Semitendinosus	34.9	12.9	20.9	7.9
Leg				
Gastrocnemius	49.3	20.6	29.7	16.1
Plantaris	10.7	1.6	4.9	2.3
Soleus	19.7	7.0	9.1	5.6
Popliteus	4.0	4.1	3.3	2.3
Tibialis posterior	4.2	2.3	3.5	2.0
Flexor digitorum fib/hal	14.7	7.1	11.2	3.7
Flexor digitoru tibialis/long	4.3	3.2	4.5	6.1
Peroneals (longus and brev)	9.9	5.3	11.3	5.9
Tibialis anterior	15.6	7.0	13.5	8.6
Extensor digitorum longus	6.7	3.6	4.3	2.7
Extensor hallucis longus	6.6		1.9	1.2
Abductor hallucis longus	1.7		5.2	

Blank: not available.

**Table 4:** Lengths and indices.

Bones	Patas male (mm)	Vervet male (mm)	Vervet female (mm)
Clavicle	58	54	41
Humerus	190	134	110
Radius	201	125	107
Femur	219	165	132
Tibia	215	149	129
Foot	167	130	118
Hand	114	85	76
Indices			
Intermembral	90	83	83
Brachial	106	93	97
Crural	98	90	98
Humerofemoral	87	81	83
Claviculohumeral	31	40	37

Female patas not available.
